# Association of Inflammatory Periapical Lesions with Maxillary Sinus Abnormalities: a Retrospective Cone-Beam Computed Tomography Study

**DOI:** 10.30476/DENTJODS.2021.87286.1254

**Published:** 2021-12

**Authors:** Saeede Zadsirjan, Mahnaz Sheikhi, Ali Dakhilalian, Mojgan Feli

**Affiliations:** 1 Dept. of Endodontics, Dental School, Shahid Beheshti University of Medical Science, Tehran, Iran; 2 Torabinejad Dental Research Center, Dept. of Oral and Maxillofacial Radiology, School of Dentistry, Isfahan University of Medical Sciences, Isfahan, Iran; 3 Dentist, Tehran, Iran; 4 Postgraduate Student, Dept. of Endodontics, Dental School, Shahid Beheshti University of Medical Science, Tehran, Iran

**Keywords:** Cone-beam computed tomography, Maxillary sinus, Periapical periodontitis, Periostitis, Polyps

## Abstract

**Statement of the Problem:**

Odontogenic infections such as periapical lesions (PLs) can cause changes in the adjacent tissues. Infection of the maxillary posterior teeth can be easily transmitted to the maxillary sinus and cause changes in the maxillary sinus mucosa. Cone-beam computed tomography (CBCT) has high accuracy and sensitivity for detection of odontogenic lesions and is efficient for maxillary sinus assessment.

**Purpose:**

This study aimed to assess the maxillary sinuses for abnormalities such as mucosal thickening, polyps, and periostitis, and evaluate the periapical status of maxillary posterior teeth considering the presence of PLs, their size and distance from the sinus floor by evaluating CBCT images.

**Materials and Method:**

This retrospective, cross-sectional study evaluated the CBCT scans of 143 patients, depicting the posterior maxilla with at least one premolar or molar tooth present in this region. Sinus abnormalities (mucosal thickening, sinus polyps, and periostitis) and presence/ absence of PLs, its size, and its distance from the sinus floor were all assessed on CBCT scans. Data were analyzed using the Chi-square test in SPSS version 21 (a= 0.05).

**Results:**

PLs were observed in 31.2% of the cases. In presence of PLs, mucosal thickening was noted in 56.8%, sinus polyps in 29.6% and periostitis in 1.3% of the maxillary sinuses. All teeth with a CBCT periapical index (CBCTPAI) score of 5 were associated with sinus abnormalities; there was neither correlation between the sizes of lesions, nor their distance from the sinus floor with sinus abnormalities (*p*> 0.05).

**Conclusion:**

PLs in the posterior maxilla have a direct correlation with the maxillary sinus abnormalities. However, the size or distance of PLs from the sinus floor had no significant effect on the frequency of sinus abnormalities.

## Introduction

Following dental pulp necrosis, infection may spread into the periapical space and cause pathological changes in the periapical tissues. The interactions between the stimulants leaked out from the canal space and the host response can lead to activation of host immunity reactions. The infection at the periapical region of the maxillary posterior teeth can extend to the maxillary sinus via the blood and lymphatic vessels and cause sinus diseases [ 1 ], which may vary from a simple sinusitis to severe rhino sinusitis and even brain abscess, depending on the severity of tooth infection [ 2 ]. 

The maxillary sinusitis (MS) is a common condition with significant health-related complications [ 3 ]. Traditionally, odontogenic MS has been stated to account for 10%-12% of all cases of MS, an analysis of recent data indicates a much higher prevalence of 30%-40% [ 4 ]; 83 percent of these are reportedly due to apical and marginal periodontitis [ 5 ]. Recent studies have documented correlations between dental pathology and radiographic signs of sinusitis, predominantly mucosal thickening of the Schneider membrane [ 6 - 9 ].

Identifying the relationship between odontogenic and sinusal pathologies is essential to establish the correct diagnosis and management of the patient [ 10 ]. In the otolaryngology literature, odontogenic MS is reported to be underdiagnosed and often overlooked, leading to persistent symptoms in patients and the failure of medical and surgical sinusitis therapy[ 4 , 11 ].

Radiographs are effective instruments in diagnosis of periapical changes and MS abnormalities [ 12 ]. Radiographs, however, are 2-dimensional (2D) representations of 3-dimensional (3D) structures, making it particularly difficult to determine the relationship of roots and periapical lesions (PLs) with the MS floor [ 13 , 5 ]. Cone-beam computed tomographic (CBCT) imaging is capable of producing high-quality 3-dimensional images using a reduced dose of radiation and has a lower cost compared to multi-slice computed tomographic imaging [ 14 ].CBCT imagery enables the detection of changes in the maxillary sinus and their possible causes [ 7 ].

This is an ideal examination tool for assessing patients who have both dental and sinus complaints [ 15 ], and is considered as a useful technique to evaluate the relationship between maxillary sinus and adjacent teeth [ 4 ].

This retrospective, cross-sectional study aimed to assess the maxillary sinuses for abnormalities such as mucosal thickening, polyps and periostitis, and to evaluate the periapical status of maxillary posterior teeth in terms of presence of PLs, their size and distance from the sinus floor by evaluating CBCT images obtained from an archived collection. 

## Materials and Method

This retrospective, cross-sectional study evaluated 143 CBCT scans representing at least one maxillary posterior quadrant with one premolar or molar tooth present in the region (260 maxillary sinuses). The CBCT scans were retrieved from the archives of a private oral and maxillofacial radiology clinic from March 2011 to March 2016. The CBCT images had been taken for purposes not related to this study (such as implant treatment and trauma cases). 

All CBCT scans were obtained by Orthophos XG 3D (Sirona Dental System Inc.) imaging system with a flat-panel PST detector and the following exposure settings. The maximum voltage was constant for all images, equal to 85 kVp. The amperage varied between 14 to 35 mA, depending on the size of patient. The field of view (FOV) was 8 × 8 cm and the isotropic voxel size was 0.288 mm. The exposure parameters were adjusted according to the size of patient and the manufacturer’s instructions. All CBCT scans obtained in the respective center during the aforementioned time period were evaluated and those that met our eligibility criteria were selected until the sample size was reached (convenience sampling). The images were observed on a 21-inch EIZO Medical LCD monitor with 1600 x 1200-pixel resolution using GALAXIS Viewer version 1.944 software (ID2; SICAT GmbH & Co.KG). The contrast and brightness of images were adjusted by the software processing tools for best-quality display of images. 

The inclusion criteria were maxillary CBCT scans that had optimal quality for diagnostic purposes, at least one fully erupted premolar or molar tooth with mature root had in the right or left maxillary quadrant and, showing no sign/symptom of non-odontogenic acute sinusitis, such as air-fluid level or mucosal thickening in all sinus walls. 

CBCT scans that requested because of trauma or developmental anomalies or showed an orthodontic retainer, skeletal deformities, suspected cases of tumors in the maxillary sinus, pansinusitis and history of previous surgery and related dental procedures were excluded. In addition in case of possibility of any errors in measurements when assessing the sinus and PLs, the sample would be excluded right away.

### Maxillary sinus assessments

**Figure 1:**
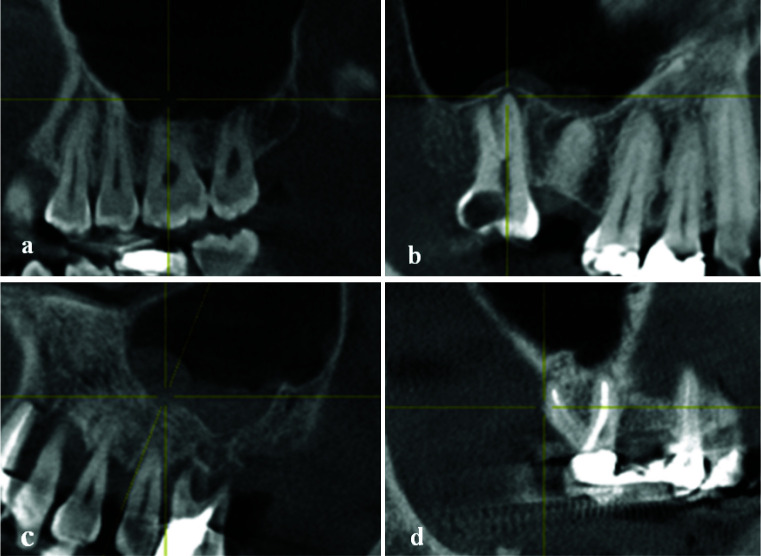
Changes in maxillary sinus observed in Cone-beam computed tomography (CBCT) imaging, **a:** Absence of alteration, **b:** Mucosal thickening, **c:** Sinus polyp, **d:** Periostitis

The findings obtained by evaluation of the maxillary sinus images were classified as follows (Figure 1) [ 12 , 16 ]. 

### Normal

The sinus space was completely radiolucent, the cortical walls were sound and intact, and there was no mucosal thickening. 

### Mucosal thickening

In some areas, the cortical bone was lost and the soft tissue density could be observed. Mucosal thickening was obvious (>1mm) and followed the bony wall of the sinus.

### Sinus polyps

Some polypoid and lobulated areas with observable soft tissue density without cortical bone were noted while the sinus floor was sound and intact.

### Periostitis

Uniform opaque areas of layered thickening in the cortical bone of the sinus floor were noted superior to a radiolucent area related to tooth apex.

**Figure 2:**
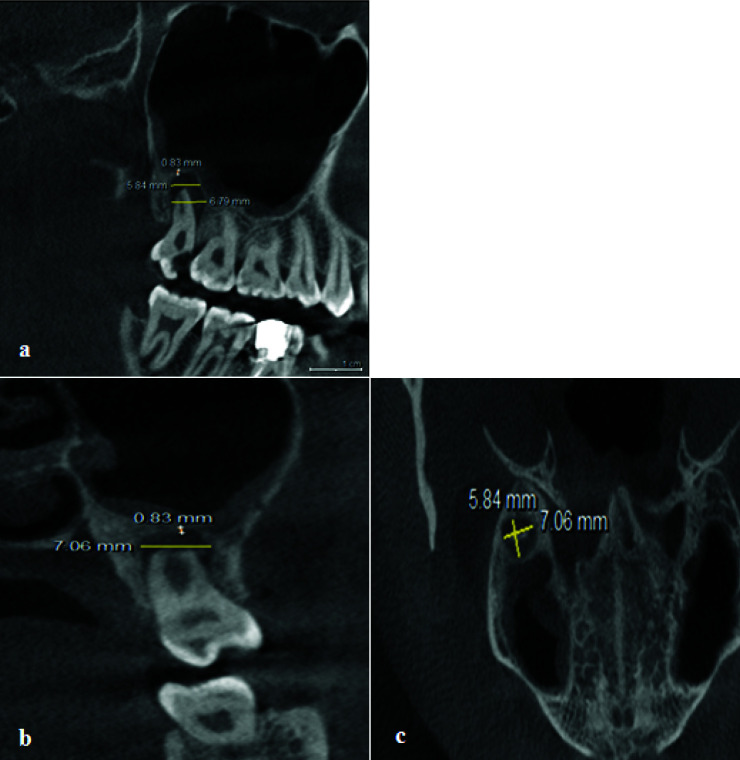
**a:** Sagittal view, **b:** Coronal view, **c:** Axial view

The CBCT images were evaluated three-dimensionally, and four measurements were made including sagittal (mesiodistal), coronal (buccolingual), and axial (mesiodistal and buccolingual). The maximum extension of lesion was recorded as the reference size and scored using a 6-point scoring system (0-5) as follows (Figure 2) [ 17 ].

### Assessment of periapical status

The teeth with a hypo dense PLs were recorded. The size of the PLs was measured using CBCT periapical index (CBCTPAI) as (0) intact periapical bony structure, (1) diameter of periapical radiolucency: 0.5-1 mm, (2) diameter of periapical radiolucency: 1-2 mm, (3) diameter of periapical radiolucency: 2-4 mm, (4) diameter of periapical radiolucency: 4-8 mm, and (5) diameter of periapical radiolucency: > 8 mm. 

In teeth with multiple PLs, the lesion with the most severe pathological condition was recorded. 

The lesions were divided into three groups based on their distance from the sinus floor as 0 mm (the lesion was attached to the maxillary sinus floor), 0-2 mm distance and > 2 mm distance.

Moreover, the maxillary sinuses were divided into two groups based on presence/absence of PLs in the posterior maxilla as (1) with at least one PL in teeth adjacent to the sinus, and (2) no tooth with PL. All images were observed by an oral and maxillofacial radiologist. In order to assess the intraobserver agreement, first 20% of the primary sample (56 images) was observed by the observer twice with a three months interval.

### Statistical analysis

Data were analyzed using SPSS version 21 (SPSS Inc., IL, USA). The prevalence of sinus pathologies was calculated in presence/absence of PLs. The results were analyzed using the Chi-square test and the correlation coefficients. The intraobserver agreement was evaluated using the kappa coefficient, and the level of significance was set at α=0.05.

## Results

The kappa coefficient of intra-observer agreement was 0.95 (*p*< 0.001) and indicated high intraobserver agreement in assessment of the variables. The CBCT scans were first divided into two groups with/without PLs. As shown in Table 1, 260 CBCT scans were evaluated; out of which, at least one PL was present in 81 cases (31.2%), while, 179 cases (68.8%) did not have any PLs. In other words, the frequency of PLs in our study population was 31.2%. The frequencies of maxillary sinus abnormalities associated with PLs, size (CBCTPAI), and distance between the upper PLs edge and the maxillary sinus floor are shown in Table 1. Most maxillary sinus abnormalities were associated with the presence of at least one tooth with a PL (*p*< .001) (Table 1). Of the 81 maxillary sinuses associated with PLs, 71(87.7%) had maxillary sinus abnormalities. The frequency of mucosal thickening in the study population was found to be 35%. In the group of maxillary sinuses with abnormalities (n= 81) that had at least 1 tooth with a PL, the most frequent abnormality was mucosal thickening (56.8%). 

**Table 1:** Frequency (%) of Maxillary Sinus (MS) Abnormalities Associated to the Presence, Size of Periapical Lesions (cone-beam computed tomographic periapical index) and Distance between the Upper Edge of the Periapical (PA)Lesion and Maxillary Sinus Floor

Radiograph factor		Normal MS	Maxillary Sinus abnormalities			*p* Value
			Mucosal thickness	Sinus Polyp	Periostitis	
PA Lesion	Absent (179)	125 (69.8%)	45 (25.1%)	8(4.5%)	1 (0.6%)	<0.001
	Present (81)	10 (12.3%)	46 (56.8%)	24(29.6%)	1 (1.3%)	
	1	4 (40%)	6 (14%)	9 (37.5%)	1 (100%)	0.464
CBCT	2	3 (30%)	8 (18.6%)	3 (12.5%)	---	
PA	3	2 (20%)	16 (37.2%)	5 (20.8%)	---	
Score	4	1 (10%)	9 (20.9 %)	5 (20.8%)	---	
	5	---	4 (9.3%)	2 (8.3%)	---	
Distance	0m	1 (10%)	11 (23.9%)	9 (39.1%)	1 (100%)	0.084
Between upper PA Lesion edge and MS floor	> 0 to < 2	5 (50%)	17(37%)	12(52.2%)	---	
	> 2m	4 (40%)	18(39.1%)	2 (8.7%)	---	

The frequency of sinus polyps in the study population was 12.3%. This rate was 24 out of 81 cases with PLs (29.6%). Periostitis was noted in 2 out of 260 cases (0.77%), whereas, it was detected in only 1 out of 81 cases with PLs (1.3%). The maxillary sinus abnormalities were noted in 78 cases out of 81 cases with PLs. In 3 cases, the PL boundaries could not be well identified and thus, the size of lesion was not reported due to the risk of errors. Thus, these 3 cases were excluded from this analysis. There were no significant differences between teeth according to CBCTPAI scores 1, 2, 3 and 4 and the presence or absence of maxillary sinus changes (*p*= 0.464) (Table 1). Nonetheless, it might be stated that presence of PLs > 8 mm would be associated with sinus abnormalities. The results showed that approximation of the PL to the sinus floor had no significant effect on the occurrence of sinus abnormalities. The Chi-square test revealed no significant correlation between the maxillary sinus abnormalities and the distance of PLs from the sinus floor on CBCT scans (*p*= 0.084).

## Discussion

In the present study, we found most maxillary sinus abnormalities were associated with the presence of at least one tooth with a PL (81 maxillary sinuses associated with PLs, 87.7% maxillary sinus abnormalities), the most frequent abnormality was mucosal thickening (56.8%) that followed by sinus polyps (29.6%) and periostitis (1.3%).

The use of CBCT imaging in dentistry has made 3D studies of maxillofacial structures possible and has contributed significantly to the identification of PLs and the evaluation of their impact on complex clinical condition [ 20 - 23 ]. CBCT imaging is considerably more sensitive than conventional radiographs and is extremely useful in investigating the involvement of MS associated with periapical infections [ 7 , 13 , 5 , 22 ]. 

Nascimento *et al*. [ 10 ] evaluated the integrity of the maxillary sinuses adjacent to PLs and reported the accuracy, sensitivity and specificity of CBCT images to be low, good and variable, respectively; low accuracy of CBCT in their study was attributed to the low inter-observer agreement and noisy images. The main limitations of CBCT include higher scatter radiation compared with medical CT as well as metal artifacts, commonly caused by metals and amalgam restorations and less commonly caused by root filling materials and dental implants [ 23 ]. In this study, images with artifacts were excluded.

At present, the CBCT systems can be divided into two groups of small (dental and regional) or large (ortho or facial) scale based on the size of FOV. The voxel size in systems with a small FOV is small (0.2-0.1 mm); thus, such systems have higher resolution and are more suitable for endodontic applications [ 24 ]. Therefore, in this study, a CBCT system with a small FOV was used to better visualize the anatomical structures in greater details. Shanbhag *et al*. [ 5 ] showed that CBCT is an efficient imaging modality for detection of periapical disease and sinus mucosal thickening; this statement has also been confirmed by some other studies [ 10 , 25 - 28 ], as well as the current study. 

Mucosal thickening is an inflammatory reaction characterized by the hyperplasia of the maxillary sinus mucosal epithelial cells [ 29 ]. In general, mucosal thickening is the most common maxillary sinus abnormality with a prevalence rate of 37% to 62% [ 8 ]. Controversy in the reported prevalence rates can be due to two reasons including different diagnostic criteria employed by different studies, and no consensus reached on minimal mucosal thickness to be considered normal; the minimal pathological thickness is believed to be 1-3 mm. 

According to Inusua *et al*. [ 25 ], sinus mucosal thickening on CBCT scans is greater than that detected by histological analysis. Thus, thicknesses > 1 mm are considered as pathological mucosal thickening on CBCT scans. In our study, thicknesses > 1 mm were considered as mucosal thickening and accordingly, the frequency of mucosal thickening in our study population was found to be 35%; such a low rate was due to the set inclusion criteria, since we tried to eliminate abnormalities with non-odontogenic origin. 

The current results revealed pathological changes in 87.7% of the maxillary sinuses in presence of PLs in the posterior maxilla, which was in agreement with the results of Shanbhag *et al*. [ 5 ] and Lu *et al*. [ 9 ]. However, Nunes *et al*. [ 12 ] demonstrated that 64.3% of maxillary sinus abnormalities were related to PLs. Such a controversy in the results can be attributed to different diagnostic criteria since Nunes *et al*. [ 12 ] considered mucosal thickness > 3 mm to be pathological. In this study, of 260 CBCT scans, 91 showed mucosal thickening; out of which, 46 were associated with PLs. In other words, 56.8% of patients with PLs in the posterior maxilla had mucosal thickening. This rate was 25.1% in patients without PLs, which indicates the positive correlation of mucosal thickening and presence of PL. This finding was in agreement with the results of a systematic review by Eggmann *et al*. [ 28 ] who showed a significant association between mucosal thickening and presence of PLs. Block *et al*. [ 27 ] reported that 50% of cases with sinus mucosal thickening in their study had a carious tooth. They re-evaluated 30 patients after endodontic treatment and found that the frequency of mucosal thickening decreased compared with the baseline preoperative rate, which indicates the presence of a significant correlation between mucosal thickening and odontogenic infection. 

The maxillary sinus polyp is another inflammatory reaction of the maxillary sinus, characterized by areas of dense folded crypts in the sinus mucosa, which can be visualized on radiographs. It has a prevalence of 6.5% to 19.4% [ 30 ]. In our study, the frequency of sinus polyps was 12.3%. Of relevant previous studies, only Nunes *et al*. [ 12 ] assessed the correlation of PLs and sinus polyps, and stated that of the maxillary sinus abnormalities related to PLs, polyps had the highest frequency after mucosal thickening (23%). This rate was 24 out of 81 cases with PLs (29%) in our study. However, of 179 maxillary sinuses without PLs, it was only noted in 8 cases (4.5%), which indicate a significant reduction. 

Periostitis of the maxillary sinus is characterized by homogenous, dense and radiopaque areas in the maxillary sinus floor, detected on radiographs. In the study by Nunes *et al*. [ 12 ], periostitis was noted in 5 out of 92 cases with PLs (5%), whereas, in our study, it was detected in only 1 out of 81 cases with PLs (1.3%). Thus, due to sample size limitation, a definite conclusion cannot be drawn regarding the presence of a significant correlation. 

The size of PL on radiographs can indicate the progression of lesion. Thus, this study assessed the possible correlation of size of PLs and the maxillary sinus abnormalities. The periapical index and CBCTPAI are commonly used to study the size of PLs. The periapical index proposed in this study (CBCTPAI) was developed on the basis of criteria established from measurements corresponding to periapical radiolucency interpreted on CBCT scans [ 17 ].

This process decreases the observer errors in measurements compared with 2D radiography [ 17 ]. In this study, the CBCTPAI was used considering the high accuracy of CBCT for this purpose. Nunes *et al*. [ 12 ] also used CBCTPAI and found no significant correlation between the size of lesions and sinus abnormalities. In our study, the size of lesions had no significant correlation with the maxillary sinus abnormalities. Nonetheless, it may be stated that presence of PLs > 8 mm would be associated with sinus abnormalities. Lu *et al*. [ 9 ] used periapical index in their study and reported sinus abnormalities in all patients with acute PLs; however, they did not find a significant association between the size of lesions and sinus abnormalities. Thus, both of the abovementioned studies support our findings. 

Approximation of the PL to the maxillary sinus floor may be a potential factor stimulating the sinus mucosa. In this study, the effect of distance between the upper limit of the PL and the maxillary sinus floor on sinus abnormalities was also analyzed. The Chi-square test revealed no significant correlation between the maxillary sinus abnormalities and the distance between the PL and the sinus floor. Lu *et al*. [ 9 ] and Rege *et al*. [ 31 ] discussed that the position of PL relative to the maxillary sinus had no significant effect on mucosal thickening. In contrast to our findings, Nunes *et al*. [ 32 ] discussed that maximum sinus abnormalities were noted when the distance between the PL and the maxillary sinus floor was 0 mm. In general, it may be stated that transmission of periapical infection to the maxillary sinus may be affected by factors such as the host resistance, factors causing the infection, and anatomical variations in different individuals in terms of presence and position of blood and lymphatic vessels. All these factors can yield variable results under similar conditions in terms of size and distance of PL from the sinus floor [ 32 ]. This cross-sectional study had a retrospective design. The CBCT scans were retrieved from the archives of a radiology clinic. Thus, the results should be interpreted with caution due to the absence of prior radiographic assessment and lack of information regarding the medical history of patients. Future studies should consider clinical examination of patients and evaluation of their medical records to increase the accuracy of patient selection. 

## Conclusion

Within the limitations of this study, maxillary posterior teeth association periapical radiolucent lesions had the highest frequency of sinus abnormalities. The size of a PL and close spatial relationship between the PL and the sinus was not associated with the frequency of sinus abnormalities. Accidental maxillary sinus findings on CBCT scans warrant thorough differential diagnosis. Frequently, they may be related to dental pathologies.

## Conflict of Interest

The authors declare that they have no conflict of interest.
